# Learning Potential in Narrative Writing: Measuring the Psychometric Properties of an Assessment Tool

**DOI:** 10.3389/fpsyg.2017.00719

**Published:** 2017-05-10

**Authors:** Léia G. Gurgel, Mônica M. C. de Oliveira, Maria C. R. A. Joly, Caroline T. Reppold

**Affiliations:** ^1^Research Laboratory in Psychological Assessment, Federal University of Health Sciences of Porto AlegrePorto Alegre, Brazil; ^2^Postgraduate Program in Human Development and Health Processes, University of BrasíliaBrasília, Brazil

**Keywords:** learning, validation studies, adolescent, software validation, psychological assessment

## Abstract

**Objective:** The Computerized and Dynamic Writing Test (TIDE) is designed to examine the learning potential of adolescents in narrative writing. This was a validation study of the TIDE based on its internal structure. Learning potential is responsible for cognitive modifiabilty according to the Theory of Cognitive Structural Modifiability (CSM) developed by Feüerstein.

**Method:** Included 304 participants between 10 and 17 years of age from schools in the South of Brazil. The data collection involved student groups that were divided according to age and school grade. Each participant reponded to the TIDE for an average of 50 min in the school's computer lab. The participants' selection criteria were: being regularly enrolled in the fifth to eighth grade and providing an informed consent form signed by a responsible caregiver.

**The exclusion criteria included:** neurological problems, having been held back in school for two or more years, not cooperating, not completing the test for any reason and physical conditions impeding the assessment.

**Results:** The Kendall test indicated agreement between two evaluators, who corrected the participants' first and second texts that resulted from applying the TIDE. The TIDE is divided into three modules. Factor analysis was applied to the first module (pre-test), which revealed a division in two factors, and to the second module (instructional module), which was divided in three factors. The reliability of the TIDE items was verified using Cronbach's Alpha with coefficients >0.7. The analysis of the third module (post-test) was based on McNemar's Test and showed statistically significant results that demonstrated an evolution in the participants' learning potential.

**Conclusion:** The TIDE proved to be valid and is considered a relevant tool for speech, language, hearing, psychological and educational assessment. The original nature of the tool presented here is highlighted, based on the dynamic assessment method, offering data on a narrative writing learning method as well as its possible adaptation to other contexts and languages. In addition, the computer-based nature of the tool is emphasized, enabling its more precise application and analysis of participant performance, in addition to its lower cost, reduced application bias and ability to test more than one person simultaneously.

## Introduction

Interest in dynamic and assisted assessment arose around 1960, based on the development of interactive evaluation procedures. Dynamic assessment is a cognitive assessment modality developed under the influence of Vygotsky's Sociocultural Theory and the work of Reuven Feuerstein in the 1970s (Sternberg and Grigorenko's, 2002; Camilleri and Law, 2013). This approach promotes assessment combined with mediation with the aim of analyzing participants' learning potential in a specific achievement task. The theory implies that assessment and teaching should be integrated to broadly understand the abilities of the participants being assessed, exploring their potential and observing changes in their performance during the task (Alavi and Taghizadeh, 2014). This assessment modality presupposes the plasticity of human learning, allowing for variations in how subjects think, as well as alternative diagnostic methods for participants who do not perform well on conventional norm-referenced tests. Alternative explanations are sought for weak learning and problem-solving performances, as well as greater understanding of the participants' difficulties. Therefore, the assessment is based on the instructions given and interactions that take place in the course of the assessment in order to gain a deeper understanding of the participants' task response process (Vygotsky, 1998a,b; Enumo, 2005; Fazlollahi et al., 2015). This type of assessment intervention is also called a “mediated learning experience” (Pena et al., 2014). Learning potential is responsible for cognitive modifiabilty according to the Theory of Cognitive Structural Modifiability (CSM) developed by Feüerstein (Feuerstein et al., 1979). It is quantified by instruments that can predict changes in initial ability patterns compared to those observed after the trainng and refers to the difference in performance between aided and unaided learning. For the use of these instruments to be appropriate, it is necessary that they have psychometric studies to search for internal structure validity (Davidson et al., 2016).

When comparing the static and dynamic assessment modalities, it is observed that the former focuses on the results the participants obtain and the profile these results produce in a standardized and structured manner. Static assessment modalities are more objective than dynamic assessment modalities and better assess participants with average performance levels. On the other hand, dynamic assessments are able to assess changes based on mediations, interaction and feedback, interpreting the results by focusing on cognitive modifiability (Linhares et al., 2006). Such assessments consider the effects of the participant's culture, linguistic differences and performance on the test itself. It is a valid modality for language tests and for increasing scores in statistical tests (Jacobs, 2001). In view of these attributes, dynamic procedures have been used more frequently since the 1970s. At the same time, there has been increasing criticism of static tests, drawing a contrast between the two assessment methods (Sternberg and Grigorenko's, 2002; Camilleri and Law, 2013).

More specifically, dynamic tools are considered important resources for assessing the writing performance of children and adolescents (Dias and Enumo, 2006). Writing assessment has been described and accomplished in various manners, including formal methods, such as standardized tests and informal methods, such as qualitative observation techniques. This choice tends to be guided by the evaluator according to the participants' needs and the context in which the test takes place (Wolfe et al., 2016). Dynamic writing assessment techniques are in line with the proposals of Wardle and Roozen (2012) and Yancey (1999). According to these authors, the future of writing assessment tends to be more expansive in order to fully capture participants' learning of the writing process, including understanding why participants succeed or fail in tasks related to the construct. Nevertheless, most studies still focus on the use of static resources and tests whose scores are based on school curriculum (Wilson et al., 2016). In this context, this study aims to seek evidence of validity of the Computerized and Dynamic Writing Test (TIDE) based on its internal structure and to describe the response process in the Brazilian context. This test is based on the dynamic assessment method, which aims to assess adolescents' learning potential in narrative text writing. The search for evidence of validity based on the internal structure is the phase in which the correlations between the items and subtests are established (using factorial analyses, for example) including evidence of reliability (American Educational Research Association (AERA), American Psychological Association (APA), National Council on Measurement in Education (NCME), 2014).

In general, quantitative and qualitative forms are included in writing assessments, whether they are based on the school curriculum or not, considering the dimensionality of the written production with regard to the students' quality and productivity (Kim et al., 2014). In this sense, there is a need to develop and seek evidence of the validity of tools that identify the characteristics of the students' writing, such as the development of sentence construction, grammar and punctuation, as well as structural aspects, such as cohesion and logical sequence (Wilson et al., 2016). The number of writing assessment instruments in the literature is limited, especially for adolescents. Existing tests do not assess the construct comprehensively (including at the word, sentence and paragraph levels) with micro- and macro-structural grammatical and orthographic aspects. In addition, the tests present little or no evidence of validity (Richards, 2015). Therefore, studies seeking evidence of validity (as described in this article) are important to changing this reality and producing techniques and quality assessments that allow for appropriate decision making about assessments and intervention programs (Plake and Wise, 2014).

It is important to use instruments that assess the writing process and pay attention to the strategies the subjects use throughout the production (Flower and Hayes, 1981; Richards, 2015). To meet this need, TIDE (Joly and Schiavoni, 2013) was organized according to the written structure production described by Hayes and Flower (1980), considering the processes of planning, translating and reviewing. In a preliminary psychometric study seeking evidence of the internal validity of this tool, statistically significant differences were found between the number of words and school performance in Portuguese, considering the text at the beginning and the end of the task. The authors highlighted the need for further studies using the tool under analysis (Joly et al., 2015). TIDE stands out when compared to other static or dynamic writing assessment instruments in the literature, as it allow specific recommendations to be made to the student/respondent about possible measures to improve his/her performance, instead of merely describing that performance (Sternberg and Grigorenko's, 2002). It also evaluates adolescents' narrative writing skills and analyzes the subject's learning potential.

## Materials and methods

### Participants

The number of study participants was calculated to comply with the “items/subject index” criterion used for sample calculations when factor analyses are required (Hair et al., 2006). The sample was comprised of 304 participants, who were chosen at the public and private schools that participated in the research. The schools were invited to participate on a convenience basis and were located in the Central, Eastern and Northern regions of Porto Alegre, RS, Brazil. The study participants were fifth to eighth graders, the largest proportion of whom was in the seventh grade (37.1%). The participants' age ranged from 10 to 17 years and 43.2% were 12 or 13 years old. In terms of gender, 64.5% of the sample was female and only 8.2% came from private schools. In total, 34.6% of the responsible guardians/caretakers considered the adolescents' performance to be good while 33.9% reported the presence of problems related to attention.

The Brazilian educational system consists of early childhood education, primary education and secondary education. According to the school data, almost half of the participants started school at 6 years of age (48.8%), 82.1% attended childhood education and 9.1% had reading or writing problems, according to their parents and/or caregivers (who completed questionnaires with information on the participants). The participants' selection criteria were: being regularly enrolled in the fifth to eighth grade and providing an informed consent form signed by their responsible caregivers. The exclusion criteria included: neurological problems, having been held back in school for two or more years, not cooperating, not completing the test for any reason and physical conditions impeding the assessment.

### Material

The goal of the TIDE (Joly and Schiavoni, 2013) is to assess the learning potential of primary education students in the production of written narrative texts. It was constructed based on the dynamic assessment principles of learning potential, in accordance with Sternberg and Grigorenko's (2002) theoretical premises and Hayes and Flower (1980) theoretical structure of written production. Its internal structure consists of a pre-test, instruction module and post-test. In the pre-test, the participant provides information, such as name, grade and age. The test then starts with instructions on the proposed task: the writing of a narrative text on the topic: “Hero for a day.” The participant is then asked to read his/her text and make any corrections that he/she deems necessary. No hints are provided to aid the respondent's performance.

The instruction module, in turn, supposes that strategies are offered to facilitate the learning of text writing, and is based on Hayes and Flower (1980) processes. This written composition model does not describe successive stages but rather the processes involved. The first of these processes is planning, which serves to extract information on the nature of the task and on long-term memory and use it to establish a plan that guides the production of a text, including three subgroups: idea production, organization and target setting. The second process is translation, which is intended to transform the planning of the message into written sentences, which should comply with the standards of written language. The third process is reviewing, which involves evaluating the text produced and improving its quality. This process contains two sub-processes: reading and editing.

The individual receives advice on how to write a narrative text and the elements to be included, such as characters, scene, situation and a problem. He/she is asked to verify whether his/her text contains all of these elements. Questions are then asked to lead the participant to reflect on his/her written construction. He/she is expected to answer the questions and revise the text if any of the elements are missing, according to his/her own judgment. The first questions focus on the characters in the story (e.g., “Who are the main characters?,” “What do they look like?”). Questions are then asked about the scene, situation, problem faced, response to the problem, action, solution and reaction of the characters in the story. Finally, the participant is asked to revise his/her text based on final questions. Afterwards, he/she is instructed to review whether the sentences are complete, whether proper names start with capital letters, whether the words are spelled correctly and whether punctuation marks are used appropriately. The result is analyzed on a three-point Likert scale (participant did not add the answer to the question to the text, participant partially added the answer to the question to the text or participant fully added the answer to the question to the text). The instruction module has 19 items.

Finally, in the post-test, the participant analyzes his/her textual output as a whole. He/she has the opportunity to reread and edit the text, if necessary, without interference or hints from the evaluator. Because it is a computerized test, the participant can access and change any item in the module that he/she deems necessary and may do so as many times as needed. The changes made while editing the text appear on the screen in a different color. In the instruction module, each element of the narrative text has a different color so that it becomes clear to the student how much he/she has altered or added to each of these elements of the narrative.

The evaluators correct the results. Automated correction is not used. The evaluators compare the initial and reformulated texts to verify the participant's performance after the instructions were provided. In the present study, two evaluators assessed the texts: a speech therapist and a psychologist, both of whom are extensively trained post-graduate students The evaluation consists comparing the participants' initial and final texts. Four specific aspects are observed in each participant's texts (initial and final/post-intervention): (1) changes made throughout the text in relation to the context of the theme and the characters; (2) changes made in relation to the development of the text (problem situation/proposed solution/action to solve the problem/character's reaction); (3) outcome of the theme and (4) overall content. The texts are assessed on a three-point Likert scale (did not comply with the criterion, partially complied with the criterion or fully complied with the criterion). The difference between the punctuation of the initial and final texts is assessed for each participant. In this last phase, it is possible to measure the participant's proximal development zone—i.e., the difference between each participant's potential and actual performance (Vygotsky, 1988).

### Data collection procedures

The parents or responsible caregivers of the students from the participating schools were contacted to be informed about the research and to sign the Informed Consent Form. Only those students whose parents or responsible caregivers gave their informed consent participated in the research. The data collection took place in a private room at each school, in silence, without external interference, during regular class times, in groups of up to three participants and was monitored by three researchers. The groups were organized according age and school grade. The students received the instructions and took the test individually. The application of the complete TIDE (including the pretest, instruction model and post-test) took an average of 60 min.

Notebook computers with basic hardware capacity were used to support the TIDE software. After the test applications, the software automatically stored the tests in a database. At the end of each application session, a backup of the data was made on a removable disk. It is highlighted that this research did not pose risks to the participants and that individual privacy was preserved. Ethical aspects of research involving human beings were complied with. Approval was obtained from the Research Ethics Committee at the Federal University of Health Sciences of Porto Alegre, under protocol 502.515.

### Data analysis procedure

The test answers were corrected and scored by two independent evaluators who were fully trained to apply and interpret the data according to the TIDE's preset criteria, as described in the tool. Any disagreements about the corrections were resolved by consensus and joint analysis. Exploratory factor analyses with varimax rotation were used, which is an orthogonal method that minimizes the number of variables in each cluster, resulting in a clearer separation between the factors. Factor analysis was used in the pre-test and instruction modules, since this type of analysis studies the relationship between variables and provides evidence on the quality of the instrument's structure. It is used to identify the latent dimensions and underlying variables, contributing to the theoretical interpretation of the test (Hair et al., 2006). Extraction via Principal Components with the varimax rotation method was adopted, given the structure of the items being analyzed. In addition, Cronbach's Alpha was calculated to analyze the pre-test and the instruction modules to verify the tool's reliability.

McNemar's Test was used to analyze the pre-test in order to determine the participants' evolution by comparing the analyses of each participant's final and initial texts, as this test is used to analyze related samples. Kendall's Agreement Test was used to analyze the agreement between the evaluators of the texts. The search for evidence of validity based on the external criterion of age was performed using the Kruskall Wallis test for independent samples and the chi-square test or Mann-Whitney test was used for school grade and gender. Statistical analysis of the collected data was developed in the Statistical Package for Social Science (SPSS), version 19.0.

## Results

The Kendall Test was applied initially to verify the agreement between the two evaluators' assessments, showing appropriate results that indicate the similarity between the researchers' analyses of the participants' texts. No correlations were found between the evaluators' scores of the participants' texts. The results were as follows: question 1: tau = 0.70 (*p* < 0.01); question 2: tau = 0.74 (*p* < 0.01); question 3: tau = 0.69 (*p* < 0.01); question 4: tau = 0.92 (*p* < 0.01). Based on this information, the items and correction protocol were considered to be sufficiently clear, permitting the uniformity of the process. For the purpose of analysis, we used the scores of Evaluator #2.

Factor analysis was subsequently applied to obtain evidence of validity based on the internal structure of the proposed tool. Therefore, the following steps were followed separately for the pre-test and instruction modules: (1) analysis of the conditions for the factor analysis (using the Kaiser-Meyer-Olkin (KMO) tests and Bartlett's sphericity test) and (2) definition of the number of factors in each test module (exploratory factor analyses). For the pre-test module, the (KMO) tests and Bartlett's sphericity test were applied to indicate the appropriateness of the data for the factor analysis. The former indicates the proportion of data variance, with values closer to one indicating that the sample is more appropriate for the factor analysis. In this study, the result was 0.76, representing an average result. The latter indicates whether the matrix demonstrates correlation among the data and the result was positive (*p* < 0.001).

In Step 2 of the pretest module, the presence of two factors was observed. Factor 1 corresponded to 41.62% of the explained variance between the items and Factor 2 corresponds to 15.90%. Of all of the items, four were related to Factor 1 (situation, response, action and solution), and three were related to Factor 2 (characters, scene and reaction). With respect to the communalities (the total amount of variance an original variable shares with the others), the minimum acceptable coefficient was 0.30 (Hair et al., 2009). All items in the test had higher coefficients. The item “solution” in Factor 1 and the item “scene” in Factor 2 presented the highest communality coefficients and were shown to be the most representative of each factor. Table 1 shows the coefficients of each component and each item.

**Table 1:** **Factorial solution with factor loadings, communalities and Cronbach's Alpha for TIDE's pre-test module items (***N*** = 304)**.

**Items**	**Factors**		**Communalities**
	**1**	**2**	
Characters	0.03	0.69	0.49
Scene	0.15	0.77	0.62
Situation	0.74	0.14	0.58
Response	0.55	0.52	0.58
Action	0.73	0.22	0.59
Solution	0.85	0.04	0.73
Reaction	0.21	0.60	0.41
Number of items	4	3	

The (KMO) tests and Bartlett's sphericity test were also applied for the instruction module. The result was 0.92 for the former and the significance was *p* < 0.001 for the latter. Representing a very good result. In the instruction module, the presence of three factors was observed. In terms of variance, Factors 1, 2, and 3 presented 40.16, 8.28, and 6.73%, respectively. All items presented coefficients greater than the minimum for the communalities, ranging from 0.35 to 0.76. The items with the highest coefficients were “How is this problem solved?” in Factor 1, “How are they physically?” in Factor 2 and “What situations do they experience in the story?” in Factor 3. Table 2 displays the coefficient of each component and item.

**Table 2:** **Factorial solution with factor loadings, communalities and Cronbach's Alpha for TIDE's instruction module items (***N*** = 304)**.

**Items**	**Factors**			**Communalities**
	**1**	**2**	**3**	
Who are the main characters in the story?	0.45	0.42	0.27	0.46
What do they look like? Describe	0.08	0.73	0.15	0.57
How old are they?	0.06	0.73	0.14	0.57
What do they do? That is, what are their occupations?	0.12	0.69	0.15	0.52
What happens to them? What situations do they experience in the story?	0.39	0.48	0.20	0.43
Are there other characters in the story?	0.22	0.25	0.73	0.66
What is their relationship to the main character?	0.23	0.15	0.82	0.76
What situations do they experience in the story?	0.27	0.13	0.76	0.76
What happens in the story?	0.31	0.57	0.09	0.44
Where does the story happen?	0.59	0.39	0.18	0.54
What are these places like?	0.35	0.48	0.04	0.35
What is the situation or problem the main characters face?	0.65	0.25	0.24	0.54
What do the characters feel, think and decide to do to solve the problem?	0.72	0.17	0.21	0.59
What do the leading characters do to solve the situation?	0.73	0.13	0.17	0.58
What do the other characters do?	0.59	−0.00	0.35	0.47
How is this problem solved?	0.79	0.20	0.13	0.68
What happens at the end of the story?	0.71	0.25	0.22	0.62
What happens to the main characters and the other characters in the end?	0.68	0.24	0.13	0.54
How do the characters feel at the end of the story?	0.43	0.48	−0.03	0.42
Number of items	9	7	3	

Item reliability in the pre-test and the instruction module was verified using Cronbach's Alpha. It was observed that the TIDE coefficients were high, corresponding to 0.76 for the pretest and 0.91 for the instruction module. Internal consistency was demonstrated for the items in each of the modules tested.

McNemar's test was applied to analyze the post-test module and was used for dependent, paired samples (Hair et al., 2006). As the test requires, the answers were dichotomized and grouped under “did not comply with the criterion” and “complied with the criterion” (including “fully complied with the criterion” and “partially complied with the criterion”), thereby considering whether or not there were changes in the participants' behavior during the course of the test. Statistically significant positive results were observed, demonstrating the rejection of the hypothesis that the response percentages were the same in the initial and final texts. In other words, a relevant change was observed when considering the initial condition of the subjects' texts. Table 3 shows the results of the post-test obtained through the statistical analysis of the answers coded by McNemar's Test.

**Table 3:** **Statistical analysis results of McNemar's Test for four questions in the TIDE's post-test (***N*** = 304)**.

	**Initial**	**Final**		***x*^2^**	***p***
		**Did not meet the criterion**	**Fully or partially met the criterion**		
Question 1: changes throughout the text in the contextualization of the theme and the characters	Did not meet the criterion	35	56	54.01	<0.001
	Fully or partially met the criterion	0	213		
	Did not meet the criterion	27	32	30.03	<0.001
Question 2: development of the text. the problem situation. proposed solution. action to solve the problem and character's reaction	Fully or partially met the criterion	0	245		
	Did not meet the criterion	35	40	38.02	<0.001
Question 3: related to the outcome of the theme	Fully or partially met the criterion	0	229		
	Did not meet the criterion	231	3		<0.001
Question 4: about the content in general	Fully or partially met the criterion	0	69		

The qualitative analysis of the participants' performance revealed that, for the first aspect (changes throughout the text in the contextualization of the theme and the characters), 35.5% of the participants partially complied with the criterion in the initial text and fully in the final text, showing considerable evolution in the text production. For the second aspect (related to the development of the text, the problem situation, proposed solution, action to solve the problem and characters' reaction), a significant increase was observed in the proportion of participants who partially complied with the criterion at the beginning and fully complied at the end (21.1%). As for the third aspect (related to the outcome of the theme), the percentage of participants who still complied partially with the criterion stood out (27%). In the fourth and final aspect (regarding the content in general), a large proportion of participants continued not to comply with the criterion (76.2%). Based on the analysis of the post-test, the potential of the TIDE to diagnose the participants' difficulties was shown, as well as whether the intervention strategies resulted in better performance. These results are detailed in Table 4, which shows the descriptive statistics of the post-test results.

**Table 4:** **Descriptive statistics for assessment of the initial and final texts in the TIDE's post-test (***N*** = 304)**.

	**Initial**	**Final**	
	**Did not meet the criterion-n(%)**	**Partially met-n(%)**	**Fully met-n(%)**
**Question 1: changes throughout the text in the contextualization of the theme and the characters**			
Did not meet the criterion-n(%)	35 (11.5)	30 (9.9)	26 (8.6)
Partially met-n(%)	0	82 (27.0)	108 (35.5)
Fully met-n(%)	0	1 (0.3)	22 (7.2)
**Question 2: related to the development of the text, the problem situation, proposed solution, action to solve the problem and character's reaction**			
Did meet the criterion-n(%)	27 (8.9)	18 (5.9)	14 (4.6)
Partially met-n(%)	0	75 (24.7)	64 (21.1)
Fully met-n(%)	0	0	106 (34.9)
**Question 3: related to the outcome of the theme**			
Did not meet the criterion-n(%)	35 (11.5)	25 (8.2)	15 (4.9)
Partially met-n(%)	0	82 (27)	81 (26.6)
Fully met-n(%)	0	0	66 (21.7)
**Question 4: about the content in general**			
Did not meet the criterion-n(%)	231 (76.2)	1 (0.3)	2 (0.7)
Partially met-n(%)	0	9 (3.0)	2 (0.7)
Fully met-n(%)	0	0	58 (19.1)

In regard to the participant's age (organized in quartiles of < 12, 12, 13, and 14 years or older), it was observed that there was no significant association in the pre-test module (*x*^2^ = 6.75, linear by linear association = 1.17, and *p* > 0.005), demonstrating that there was no association between age and performace on the test. Thus, the test is configured as an instrument with a wide range of possible applications, as it does not undergo changes in the responses according to the ages of the individuals submitted to this evaluation. Similarly in the instruction module, significant changes in performance were not observed using the Kruskal Wallis test (*p* > 0.005), as the estimated score medians were similar among the age groups analyzed.

The participants' school grade, which was divided into four categories (fifth, sixth, seventh and eighth grades) also did not show any association with the subjects' performance in the pre-test module (*x*^2^ = 0.20, *p* = 0.16 and linear by linear association = 2.43). As in the instruction module, the subjects' school grade was observed to have no influence on the responses given to each item (*p* > 0.005).

Finally, the gender variable was also considered as a possible influence on the results of the present study. However, this was also not significantly related to the pre-test module or the instructional module. The pre-test module score was not associated with gender (*x*^2^ = 5.74, *p* > 0.005, and linear by linear association = 5.58) and the median instructional module score was similar for boys and girls, rendering the difference statistically non-significant.

## Discussion

Health and education researchers and professionals frequently point to the need for evaluation methods that can be adapted and used in linguistically, culturally, socially and educationally different populations. Dynamic assessment is a suitable method for this purpose. Therefore, the development and use of tests, such as the TIDE that examine participants' learning potential are considered essential (Camilleri and Law, 2013). To demonstrate the suitability of the tool, two additional aspects were addressed in this study, thus broadening its objectives: (1) Does the TIDE present appropriate psychometric properties that permit its use and are related to evidence of reliability and validity based on its internal structure? (2) Was the capacity of the TIDE to diagnose difficulties demonstrated?

The first inquiry that guided this study was intended to demonstrate the reliability and psychometric evidence of the tool and permit its use. In this sense, the initial goal was to analyze reliability by calculating the Cronbach's Alpha coefficients of the pre-test and instruction modules. The results indicated that the TIDE's Cronbach's Alpha coefficients were high, as they fell within the ideal range (> 0.70) established by Streiner and Norman (2003). The instruction module presented the highest coefficient and the tool was found to be precise overall.

Regarding the search for evidence of validity based on the internal structure, exploratory factorial analysis (EFA) was also applied to the pre-test and instruction modules. The EFA results revealed that the pre-test module was divided into two factors that support the structure of a narrative (Pinto et al., 2015). Factor 1 grouped items related to the main conflict present in the story (including situation, response, action and solution) and also characterized central issues guiding the text's development process, such as the production of ideas and the establishment of targets, in accordance with the model developed by Flower and Hayes (1981). Factor 2 included items related to the context in which the story takes place, including the characters, scene and the characters' reactions, which is also in line with the organization of ideas (Flower and Hayes, 1981). It is important to highlight that this factor presented the highest explained variance, demonstrating the relevant portion of common variance the factor extracted from the data set.

The factor loadings of the items ranged from 0.55 to 0.86, which are higher than the minimum loadings established in the literature (0.30), indicating that the TIDE items are highly relevant to assessing text production in the narrative model (Hair et al., 2009). In view of these results, it was also demonstrated that the orientation procedure in the first text production phase (and selected in the literature) was also valid (Flower and Hayes, 1981). Other studies have included this method as the basis for written production, including those by Matuchniak et al. (2014), Zheng et al. (2015), and Bruning et al. (2013).

It is highlighted that the writing process also implies metacognitive functioning, as proposed by van der Stel and Veenman (2014), which acts in an articulated manner with the text production structure, resulting in a narrative that meets the expected regulatory standards. This is the case especially due to the participant's ability to reflect and rethink his/her writing process. In this sense, the orientations and instructions provided during the TIDE promote the participant's reflection on his/her own production. Participants can develop skills related to analysis, control, planning, target setting and task revision, which take place at the beginning and the end. This process is reflective, as observed in the parameters considered in the TIDE's pre-test and instruction modules. It is also related to cognitive models, as the narrative task is sometimes considered a core cognitive action, constituting human thought. At other times, it is considered a cognitive processing model, although it is more paradigmatic and is based on more social phenomena (Genereux and McKeough, 2007).

The planning, translation and review steps of the writing model developed by Hayes and Flower's (1980) are based on the cognitive processes of text composition. Thus, the more experienced the writing participant is, the better he/she will produce objectives, storage and automation of the repertoire, in addition to reflecting on his/her writing and interpretation, including memory and motivation (Erhard et al., 2014). The internal structure of the TIDE is therefore appropriate for the assessment in question, as observed in the study conducted by Joly et al. (2015).

The EFA of the instruction module identified three factors, confirming the model proposed by Flower and Hayes (1981) for the text writing process. Thus, the TIDE can be considered to be didactically divided into three phases: planning, translation and review. The first phase is divided into the production of ideas and organization and setting of targets, while the third is divided into assessment and revision. When comparing the model by Flower and Hayes (1981) with the division into factors observed in the TIDE, Factor 1 included the items aimed at the production of ideas and setting of targets (belonging to planning) and their translation. Factor 2 included items focused on the organization of the ideas and the translation. Factor 3 included review items, which add complementary information to the text's main information (included in the items belonging to Factors 1 and 2 of the test).

For example, the model developed by Flower and Hayes (1981) does not presuppose a definitive predefined time model. Some participants take more time in the planning phase, while others put more energy into the revision phase, demonstrating variation among the participants that should also be considered and the need to make the analysis of the premise more flexible. In addition, considering the variation of the cognitive processes the participants use, the writing process should not be considered to be a strict sequence of phases, but rather a “set of optional cases.” For example, the idea production phase may need to be revised. Therefore, the writing process is flexible, despite its pre-established ideal hierarchy and despite being a goal-oriented process (Hayes and Flower, 1980). These aspects were verified in the instruction module's EFA and therefore support the classification of the TIDE's instruction module.

The tools constructed based on dynamic assessment (as shown thus far) are in line with Yancey (1999) and Wardle and Roozen (2012) as a process that values participants' learning. In this sense, the present article provides important contributions, presenting the development of a tool that enables the potential learning of writing to be analyzed. This fact can be particularly observed in the TIDE's final module (the post-test), which enabled a positive and satisfactory response to the second inquiry in this study, which was related to the potential learning and evolution in the adolescents' performance when writing the proposed narrative text.

The TIDE's post-test considers three main points: cohesion, coherence and structure. These three points are included in the four post-test questions, which provide the results of the participants' evolution, comparing the initial and final production. In addition, other relevant text production issues are considered in the post-test, such as the presentation of a beginning that permits expansion on the theme, characters and their descriptions, a scene, the resolution of the problems and an ending, thereby comprising the development of a story with a beginning, middle and end and a coherent and cohesive logical order (Genereux and McKeough, 2007; Pinto et al., 2015).

It was observed that age, school grade and sex were not factors that influenced the subjects' responses, as was the case in the pre-test module, also demonstrating the homogeneity of the sample in regard to the responses given to this module's items, as well as the instrument's stability. Further studies should be conducted to clarify and deepen the understanding of these issues, especially by using other school samples. This is due to the fact that, according to dos Santos and Befi-Lopes (2016), there is an increasing improvement in writing ability as students advance in school grades. Initially, students' writing reflects their speech, using simple syntax and orally-based vocabulary. From the fifth grade onwards, students begin to organize their sentences hierarchically, using more formality and graphic mastery and developing the linguistic and cognitive skills of written language.

The results for the four questions mentioned earlier showed statistically significant coefficients, demonstrating the appropriateness of the answers provided. Thus, it can be assumed that, among the four criteria the evaluators considered in the initial and final texts of each participant, it appears that the instructions the participants received during the course of the instruction module did not only influence the overall content. This fact can be observed in the large number of participants who did not comply with the criterion initially and continued not to comply in their final text. The remaining questions are easier to change in the text and are more open to improvements, while the content (which represents the core idea in the story) requires more effort and modification for its qualitative evolution.

In response this study's second research question (based on the analysis of the post-test), the TIDE's potential to diagnose the participants' difficulties and learning potential was demonstrated. The participants' learning potential was based on the premises of hints and instructions. It has been confirmed that the dynamic assessment method, as originated by Vygotsky (1987), enables the verification of emerging skills, developmental possibilities and the quality of mediated teaching. It also allows for the designation of the participants' proximal development zone through a more qualitative analysis, providing information and describing the participants' trajectory of written production (Poehner and Lantolf, 2013).

Regarding the final analysis of the test the TIDE proposes, no established standard exists to share the diagnosis observed in the dynamic testing. Nevertheless, Poehner et al. (2015) have commented that three manners are described in the literature: the creation of student categories, the proposal of scores and the description of student profiles. In this sense, the TIDE is scored to indicate a participant's performance based on a comparison of his/her initial and final texts, which supports the determination of his/her performance and learning potential. The TIDE also enables the respondent's profile to be analyzed through a qualitative analysis of his/her productions.

Finally, the computerized nature of the dynamic assessment of writing is discussed in order to promote reflection on the certainty and validity of this type of resource to assess adolescents' learning potential. Regarding this theme, Poehner and Lantolf (2013) showed that the first attempt to use computerized dynamic assessment was through the “*Lerntest”* tool, which was a learning test developed by Guthke and Beckmann (2000). Other tools were later developed, such as the “C-DA,” conceived by Tzuriel and Shamir (2002), which assesses cognitive aspects of mathematics learning, providing information on the student's learning. According to the authors, both tools are appropriate for computerized assessments. In this sense, the computerized assessment environment and its possible interaction are crucial points for evaluation and mediated learning activities. Also based on Vygotsky's theory of the proximal development zone, the potential for dynamic assessment based on external forms of mediation is highlighted, which can include dialogical interaction with an advisor and the use of resources, such as graphs, diagrams and models (Alderson, 2007; Poehner et al., 2015), including computers in this case.

There is a difference between the dynamic testing method developed by (Poehner et al., 2015) and the TIDE The former method interacts with participants to demonstrate when the answer is wrong through the sentence “That is not the correct answer.” On the other hand, due to its more comprehensive nature and because it proposes a freer task, the TIDE questions the participants about the text's components, teaching them the core aspects of a narrative text and the appropriate writing process in general, without any feedback on possible right or wrong answers. The main idea of the TIDE, as described by Tzuriel (2000), is to attempt to transform a students' impulsive writing style into a reflective model, granting them the opportunity to reflect on their writing process in a more conscious manner.

It is important to note that the information provided in this test is not specific to each participant and does not depend on his/her performance. In the TIDE, assessment centers on the inquiries the participants are confronted with and on the transfer of the questions asked during the instrumental module to the actual text. A participant's negative response to questions will point to topics that need to be changed, thus leading him/her to improve his/her text afterwards. Likewise, his/her positive response to questions will point to appropriate aspects of the text produced, indicating that there is no need to modify the material.

The purpose of the TIDE is to assess the potential to learn writing, using strategies that teachers would use to help students improve their narrative writing, such as a structured outline, asking them questions and encouraging editing and revising. The TIDE's purpose is to standardize this intervention in order to offer an instrument to the academic community that is capable of evaluating such issues, using technological resources.

Other authors also use digital platforms to stimulate students' narrative writing, as in the case of Malaysia (Annamalai et al., 2013), transforming the platform into space to learn narrative writing, demonstrating the participants' evolution in comparison with traditional methods. Therefore, there is a clear need for a preliminary analysis of aspects related to the participants that will enable the application of computerized writing tests, such as lack of experience, familiarity and knowledge about using this type of tool (Jeong, 2014).

Writing differences were also observed among the participants according to sex and age, with women performing better. A dichotomy was found with regard to age in that the older participants had more mature writing skills but less motivation, which appeared to decline with age (Troia et al., 2013). Therefore, new studies using the TIDE are being prepared, taking into account and analyzing data on participants and related constructs and looking for evidence of validity based on external criteria.

## Conclusion

As observed in the literature, there is still a lack of investment in the assessment of students' written expression. Nevertheless, as a result of the more widespread use of computers, mobile phones and text messages, ideas are increasingly being transmitted in written form. Therefore, precise assessment methods are important for the analysis of and early intervention in persistent writing problems, including in the composition and construction of written texts (Berninger et al., 2015). In this sense, the present article offers relevant contributions to the specialized literature, as it presents data on the process of searching for evidence of the validity of the internal structure of an innovative test to assess adolescent narrative writing. Thus, the lack of proposed tools that specifically focus on adolescents is minimized.

In conclusion, (TIDE) is an appropriate and precise tool for assessing adolescent narrative writing that focuses on this population's learning potential (particularly in Brazil), which is the focus of this study in searching for evidence of validity. The original nature of the tool presented here is highlighted, based on the dynamic assessment method, offering data on a learning method for narrative writing as well as its possible adaptation to other contexts and languages. In addition, the computer-based nature of the tool is emphasized, enabling more precise application and analysis of the participants' performance, thus lowering costs, reducing application bias and enabling the testing of more than one person simultaneously (Bauer et al., 2012). On the other hand, the tool has a limitation in that it lacks flexibility in the instructions provided throughout the assessment process, as the computerized tool is pre-programmed. The instructions do not change during the course of the test, although the authors point out the possibility of using other instructional resources for participants who are more experienced with the task (Alderson, 2007; Poehner et al., 2015).

Finally, this study is a pioneer in its field. In addition to presenting an innovative tool and evidence of the validity of its internal structural, it aims to stimulate further research on this topic. In line with the suggestions by Muniz et al. (2015), more studies are needed to investigate the relationship between the assessment of learning potential and related constructs, such as participants' educational performance and school grade.

## Ethics statement

This study was carried out in accordance with the recommendations of Research Ethics Committee at Universidade Federal de Ciências da Saúde de Porto Alegre with written informed consent from all subjects. All subjects gave written informed consent in accordance with the Declaration of Helsinki. The protocol was approved by the Research Ethics Committee at Universidade Federal de Ciências da Saúde de Porto Alegre, under protocol 502.515.

## Author contributions

LGG and CTR - contributed to the conception and design of the work, as well as the acquisition, analysis, and interpretation of data for the work. MMCO and MCRAJ - drafted the work and revised it critically for important intellectual content.

### Conflict of interest statement

The authors declare that the research was conducted in the absence of any commercial or financial relationships that could be construed as a potential conflict of interest.
